# SHP-1 mediates cigarette smoke extract-induced epithelial–mesenchymal transformation and inflammation in 16HBE cells

**DOI:** 10.1515/med-2024-0991

**Published:** 2024-07-31

**Authors:** Quan He, Shuanglan Xu, Xiaomei Ma, Yuanxia Qian, Xuzhi Lu, Weiqi Feng, Zi Chen

**Affiliations:** Department of Respiratory and Critical Care Medicine, Zhenjiang Hospital of Integrated Traditional Chinese and Western Medicine, Zhenjiang, Jiangsu, 212000, China; Department of Respiratory and Critical Care Medicine, The Affiliated Hospital of Yunnan University, The Second People’s Hospital of Yunnan Province, Kunming, Yunnan, 650021, China; Department of Pharmacy, Zhenjiang Hospital of Integrated Traditional Chinese and Western Medicine, Zhenjiang, Jiangsu, 212000, China; Department of Respiratory and Critical Care Medicine, The First Affiliated Hospital, Nanjing Medical University, 300 Guangzhou Road, Nanjing, Jiangsu, 210029, China

**Keywords:** COPD, CSE, SHP-1, EMT, inflammation, PI3K/AKT pathway

## Abstract

Src-homology region 2 domain-containing phosphatase 1 (SHP-1) is considered an anti-inflammatory factor, but its role in chronic obstructive pulmonary disease (COPD) remains unknown. Herein, overexpression of SHP-1 was utilized to explore the functions of SHP-1 in COPD models established by stimulating 16HBE cells with cigarette smoke extracts (CSE) *in vitro*. SHP-1 was downregulated in both COPD patients and CES-treated 16HBE cells. SHP-1 overexpression reinforced cell viability and significantly prevented CSE-induced cell apoptosis in 16HBE cells. Furthermore, SHP-1 overexpression greatly reversed the CSE-induced migration, epithelial–mesenchymal transition (EMT), and pro-inflammatory factor production in 16HBE cells. In addition, CSE activated the P65 and PI3K/AKT pathways in 16HBE cells, which was also reversed by SHP-1 overexpression. Our findings indicated that SHP-1 alleviated CSE-induced EMT and inflammation in 16HBE cells, suggesting that SHP-1 regulated the development of COPD, and these functions may be linked to the inhibition of the PI3K/AKT pathway.

## 1 Introduction

Chronic obstructive pulmonary disease (COPD), a chronic airway disease, is mainly characterized by lung inflammation and airway remodeling [1,2]. Exposure to toxic particles and gases, such as cigarette smoke extracts (CSE), biofuels, and air pollution, is the leading risk factor for COPD [3,4]. Currently, a complete cure for COPD remains elusive; thus, the primary therapeutic approaches focus on ameliorating symptoms and impeding disease progression [5].

Src-homology region 2 domain-containing phosphatase 1 (SHP-1) is a non-receptor protein tyrosine phosphatase expressed mostly in epithelial and hematopoietic cells [6,7]. SHP-1 has been reported to regulate inflammation and the immune response [8–10]. Lin et al. showed that steatohepatitis is ameliorated and proinflammatory cytokines were inhibited when SHP-1 was expressed ectopically [11]. In lung disease, Oh et al. found that deletion of SHP-1 aggravated Th2 cell-dominated lung inflammation through activating the IL4/IL13 pathway [12]; Zhang et al. further confirmed that SHP-1 deficiency resulted in the dysregulation of mast cells that increased Th2 cytokines and led to lung inflammation [13]; Moreover, Li et al. reported that overexpression of SHP-1 could protect mice’s lung tissues from inflammation and cell apoptosis [14]. Furthermore, a recent study found that SHP-1 has an antifibrosis effect on lung [15]. However, the role of SHP-1 in the pathology of COPD remains to be investigated.

Herein, we used CSE stimulating16HBE cells to establish a COPD model *in vitro*, and SHP-1 was overexpressed to determine whether it impacted the CSE-induced changes in biological behaviors of 16HBE cells.

## 2 Materials and methods

### 2.1 Serum samples collection

Blood samples were obtained from 10 COPD patients and 10 healthy volunteers (as normal controls) in Zhenjiang Hospital of Integrated Traditional Chinese and Western Medicine, and serum was obtained by centrifugation and stored at −80℃. All participants underwent lung function assessments. Patients diagnosed with COPD met the diagnostic criteria outlined by GOLD. Conversely, the healthy volunteers exhibited normal lung function. The exclusion criteria encompassed the following points: (1) among other respiratory ailments, bronchial asthma, tuberculosis, and bronchiectasis were present; (2) concurrent solid tumors or hematopoietic system disease; and (3) recent utilization of immunosuppressants and steroids within the preceding 2 weeks.

### 2.2 Cell culture, transfection, and induction

SHP-1 overexpression plasmid (pcDNA3.1-SHP-1, SHP-1) and vector pcDNA3.1 (NC) were generated by GenePharma (Shanghai, China). The 16HBE human airway epithelial cells were maintained at 37℃ in Dulbecco’s modified eagle’s medium with 10% fetal bovine serum (FBS). When 16HBE cells reached 70% confluence, the SHP-1 overexpression plasmid and vector pcDNA3.1 were transfected into cells using Lipofectamine 3000 for 24 h, respectively (Invitrogen, Carlsbad, CA, USA), and then used for further experiments. CSE was prepared as previously described [16]. To establish the model of COPD *in vitro*, 5% CSE was utilized to stimulate 16HBE cells for 24 h [17].

### 2.3 Quantitative real-time PCR (qPCR)

Total RNA from 16HBE cells were isolated using TRIzol reagent (Invitrogen). M-MLV reverse transcriptase kit (Invitrogen) was utilized to synthesize cDNA. qPCR was carried out on a BioRad iQ5 system (Hercules, California, USA) with SYBR Green methods. The primer sequences are provided in Table 1. The 2^−ΔΔct^ formula was utilized to determine the relative gene expression, with normalization to β-actin.

**Table 1:** Primer sequences for qPCR

Gene	Forward (5′–3′)	Reverse (5′–3′)
SHP-1	ATCACCTATCCCCCAGCCAT	CTGAGGCTGAGGACAGCAC
β-Actin	CTTCGCGGGCGACGAT	CCACATAGGAATCCTTCTGACC

### 2.4 3-(4,5)-dimethylthiahiazo(-z-y1)-3,5-di-phenytetrazoliumromide (MTT) assays

The 16HBE cells with different treatments were incubated with MTT for 4 h in the 96-well plates at 37℃. 200 µL of dimethylsulfoxide was used to dissolve the formazan crystals for 10 min in each well. Cell viability was assessed by measuring the absorbance at 540 nm.

### 2.5 Flow cytometry

For cell apoptosis detection, an Annexin V-FITC/PI apoptosis Kit (Elabscience Biotechnology Co., Ltd., Wuhan, China) was utilized. Following treatment, 16HBE cells were harvested, washed, and resuspended in 1× binding buffer. The cells were then stained with 5 µL Annexin V and 5 µL PI for 15 min and analyzed by flow cytometry using a FACScan system.

### 2.6 Scratch wound-healing assay

The treated 16HBE cells were grown on 6-well plates to reach 80–90% confluence. Scraping the monolayer cell with a 100 µL pipette tip led to the creation of a wound. Later, the detached cells in the starvation medium were washed and removed. The monolayer cell was then starved in an FBS-free medium for 2 h, followed by feeding with a medium containing 10% FBS. The scratch width was measured at 0 and 24 h using ImageJ software.

### 2.7 Transwell assay

The treated 16HBE cells were harvested and resuspended in an FBS-free medium and plated in a transwell insert at 5 × 10^5^ cells/mL, 100 µL/well. Meanwhile, 600 µL medium with 20% FBS was added to the bottom of 24-well plates. 24 h later, 4% paraformaldehyde was employed to fix the cells for 10 min, followed by 30 min of staining with crystal violet. The non-migrating cells on the transwell insert were removed with a swab. Subsequently, the migrated cells were captured and counted.

### 2.8 Enzyme-linked immunosorbent assay (ELISA)

The supernatants of 16HBE cells were collected after treatments. ELISA kits (Beyotime, Shanghai, China) were utilized to determine interleukin-1β (IL-1β), IL-6, and tumor necrosis factor α (TNF-α) levels.

### 2.9 Western blot

Serum samples and 16HBE cells were lysed with ice-cold RIPA buffer (Beyotime). 30 µg protein aliquots per lane was run on 10% dodecyl sulfate, sodium salt-polyacrylamide gel electrophoresis, and then transferred onto PVDF membranes. We then blocked the membranes with 5% non-fat milk for 1 h. Next, membranes were incubated with SHP-1 (ab124942, 1:1,000, Abcam), Cleaved-caspase-3 (ab2302, 1:500, Abcam), BAX (ab243140, 1:5,000, Abcam), BCL-2 (ab32124, 1:500, Abcam), E-cadherin (ab227639, 1:250, Abcam), α-SMA (ab223068, 1:500, Abcam), p-P65 (S536, ab278777, 1:2,000, Abcam), P65 (ab76311, 1:20,000, Abcam), Phosphoinositide 3-kinase (PI3K, ab139307, 1:1,000, Abcam), phospho-PI3K (p-PI3K, Y464, ab138364, 1:500, Abcam), AKT (ab18785, 1:1,000, Abcam), p-AKT (ab38449, T308, 1:1,000, Abcam), or β-actin (ab252556, 1:400, Abcam) primary antibodies, followed by secondary antibodies. Protein bands were developed by ECL kits (PIERCE Biotechnology, Rockford, IL, USA) and quantified using ImageJ software.

### 2.10 Statistical analysis

Three duplicates of the experiment were performed. Statistical analyses were implemented in GraphPad Prism 8.0. Results were expressed as mean ± standard deviation. The statistical difference was analyzed using the Student’s *t*-test for two groups and a one-way analysis of variance followed by a Tukey post hoc test for multiple groups. *p* < 0.05 was regarded as significant.


**Informed consent:** informed consent has been obtained from all subjects.
**Ethical approval:** Ethical approval was obtained from the Ethics Committee of the Zhenjiang Hospital of Integrated Traditional Chinese and Western Medicine.

## 3 Results

### 3.1 SHP-1 is downregulated in COPD and CES-induced 16HBE cells

**Figure 1:**
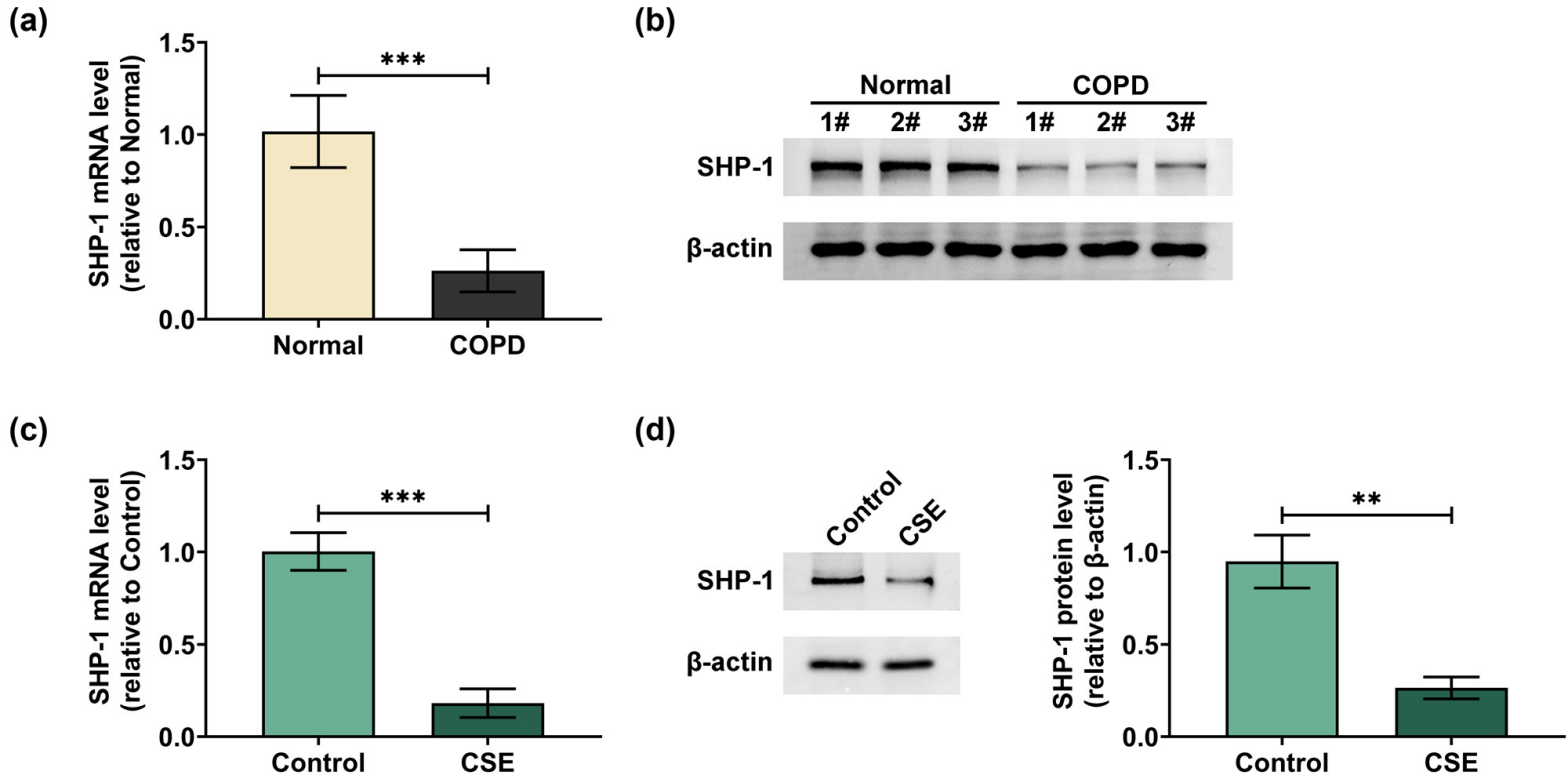
SHP-1 was downregulated in COPD and CES-induced 16HBE cells. SHP-1 mRNA and protein expression in serum samples of COPD patients and healthy controls (normal) was determined by qPCR (a) and western blot (b). SHP-1 mRNA and protein expression in 16HBE cells treated with or without CES was measured by qPCR (c) and western blot (d). **p < 0.01, ***p < 0.001.

The expression of SHP-1 was first detected in COPD serum samples using qPCR and western blot. As shown in Figure 1a and b, mRNA and protein expression of SHP-1 were lower in COPD patients’ serum compared with the normal group. Furthermore, SHP-1 expression in CSE-stimulated 16HBE cells was determined, and the results showed that mRNA and protein expression of SHP-1 were decreased in 16HBE cells exposed to CSE compared to the control group (Figure 1c and d). These data indicated that SHP-1 was lowly expressed in COPD and CES-treated 16HBE cells.

### 3.2 SHP-1 regulates CES-induced 16HBE cell proliferation and apoptosis

**Figure 2:**
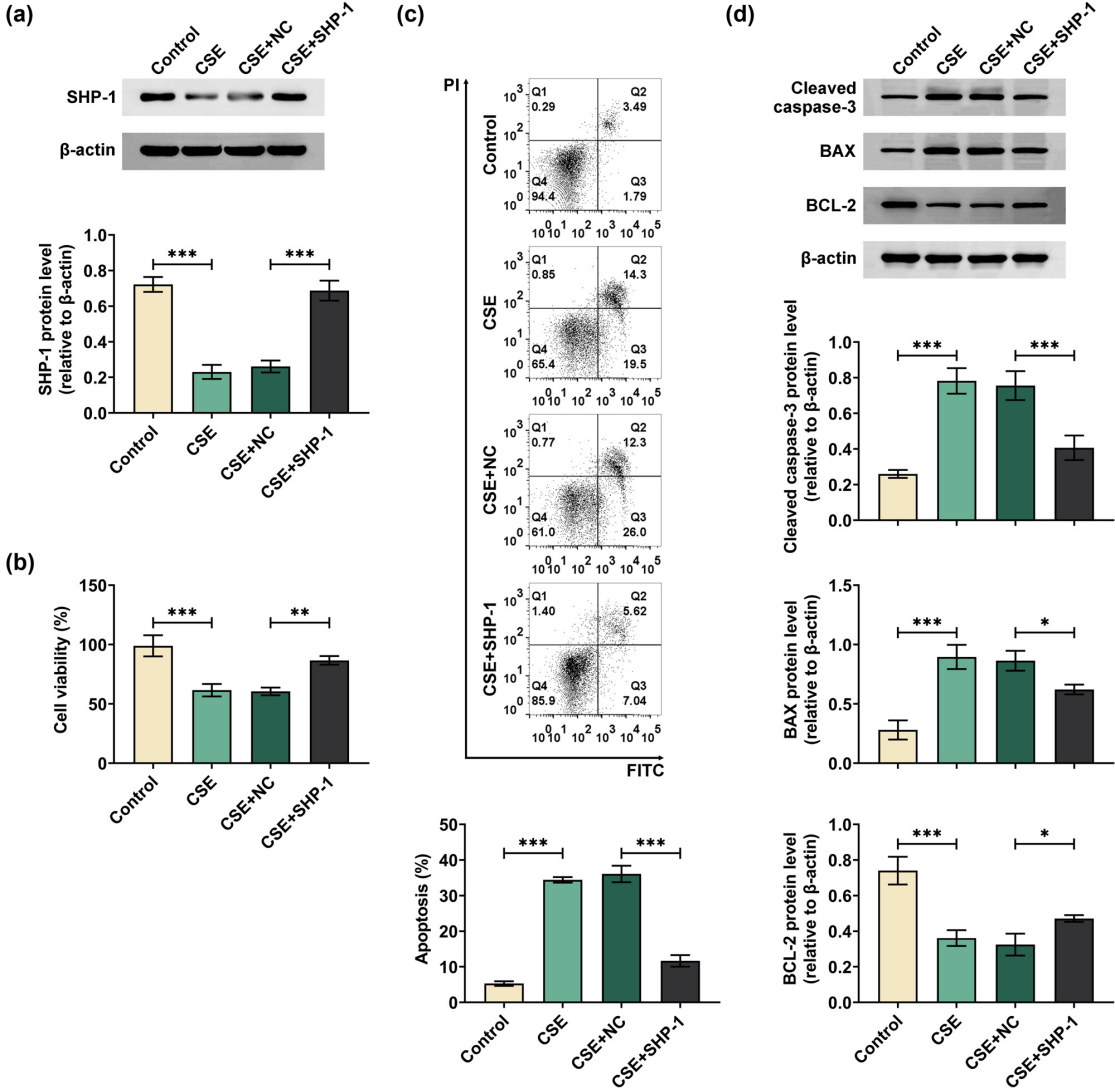
SHP-1 promoted CES-induced 16HBE cell proliferation and inhibited cell apoptosis. 16HBE cells were transfected with SHP-1 overexpression plasmid or vector and then treated with 5% CES for 48 h. (a) The transfection efficiency of SHP-1 overexpression plasmid was detected using Western blot. (b) Cell proliferation activity was assessed by MTT assays. (c) Cell apoptosis was examined through flow cytometry. (d) Protein expression of Cleaved caspase-3, BAX, and BCL-2 was analyzed by western blot. *p < 0.05, **p < 0.01, ***p < 0.001.

**Figure A1:**
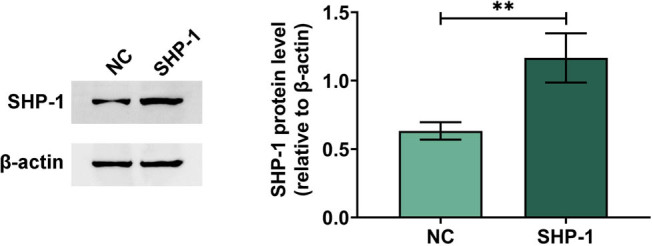
Western blot was used to analyze the transfection efficiency of SHP-1 overexpression in 16HBE cells.

To explore the role of SHP-1 in the proliferation and apoptosis of CSE-stimulated 16HBE cells, SHP-1 was overexpressed in 16HBE cells, and then, MTT assays and flow cytometry were conducted. SHP-1 overexpression was confirmed by western blot (Figure 2a and Figure A1). The results of MTT assays showed that the viability of 16HBE cell was decreased by CES stimulation, which was partly reversed by SHP-1 overexpression (Figure 2b). Flow cytometry showed an increased apoptosis rate in CES-treated 16HBE cells. However, cell apoptosis was observed to be reduced in the SHP-1 overexpressed group compared to the NC group under CES stimulation (Figure 2c). Moreover, apoptosis-related proteins were analyzed by western blot, as shown in Figure 2d, CES increased cleaved caspase-3 and BAX protein levels and decreased BCL-2 protein levels, which was reversed by SHP-1 overexpression. These data suggested that SHP-1 can promote proliferation and protect 16HBE cells from CES-induced apoptosis.

### 3.3 SHP-1 suppresses the migration and epithelial–mesenchymal transition (EMT) of 16HBE cells caused by CES

**Figure 3:**
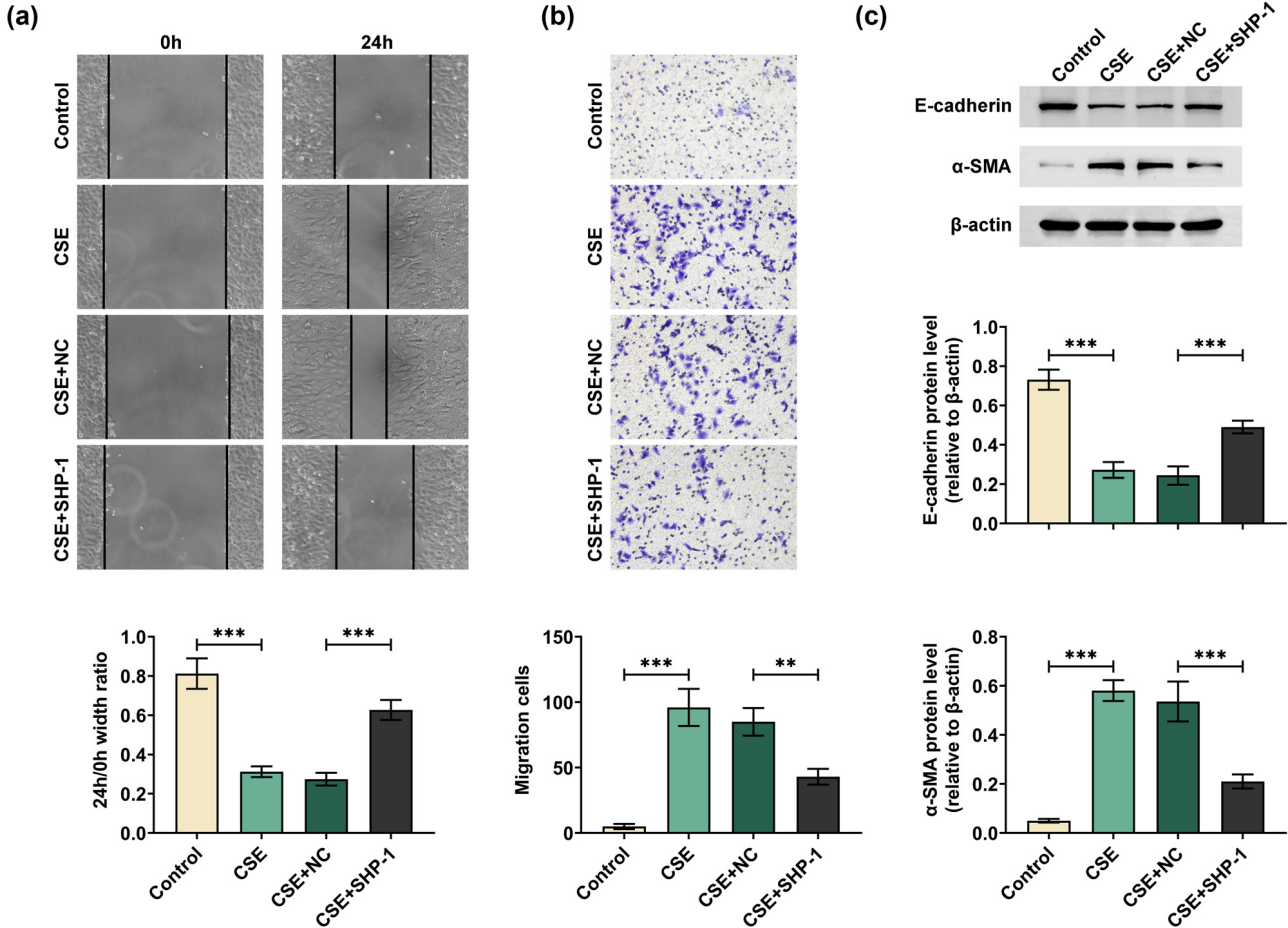
SHP-1 inhibited CES-induced 16HBE cell migration and EMT. 16HBE cells were transfected with SHP-1 overexpression plasmid or vector and then treated with 5% CES for 48 h. Migration ability of 16HBE cells detected by scratch wound-healing assay (a) and transwell assay (b). (c) Protein expression of E-cadherin and α-SMA was analyzed by western blot. **p < 0.01, ***p < 0.001.

Next, cell migration and EMT of 16HBE cells were evaluated. The results of the scratch wound-healing assay showed that the scratch width of CES-treated 16HBE cells was decreased compared to the Control group. However, the scratch width in the CES + SHP-1 group was increased compared to the CES + NC group (Figure 3a). Transwell migration assays showed enhanced migration numbers in CES-treated cells compared to the control cells, while reduced cell migration numbers were observed after SHP-1 overexpression (Figure 3b). Additionally, western blot analysis of EMT-related proteins revealed that CSE treatment resulted in a decrease in E-cadherin protein and an elevated level of α-SMA protein in 16HBE cells. Importantly, these effects were partly reversed by SHP-1 overexpression (Figure 3c). These data demonstrated that SHP-1 could inhibit CSE-induced migration and reversed CSE-induced EMT in 16HBE cells.

### 3.4 SHP-1 alleviates inflammation of 16HBE cells upon CES stimulation

**Figure 4:**
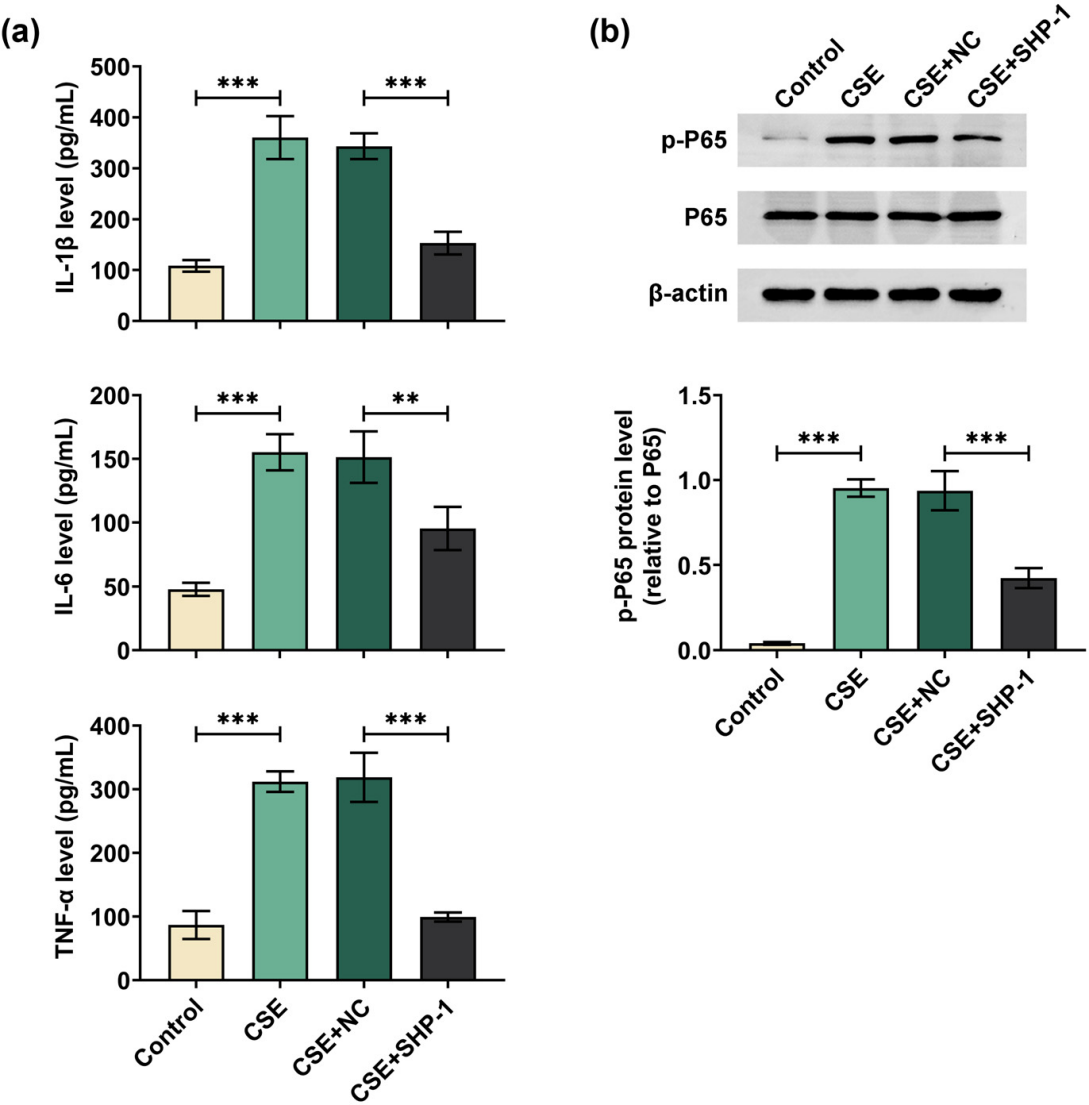
SHP-1 alleviated inflammation of 16HBE cells upon CES stimulation. 16HBE cells were transfected with SHP-1 overexpression plasmid or vector and then treated with 5% CES for 48 h. (a) The levels of IL-1β, IL-6, and TNF-α in the culture medium were detected using the ELISA methods. (b) The protein expression of phosphorylated P65 and total P65 was examined by western blot. **p < 0.01, ***p < 0.001.

Further, we evaluated whether SHP-1 affected CES-induced inflammation in 16HBE cells. The results of ELISA showed that the medium from 16HBE cells under CES stimulation exhibited elevated levels of IL-1β, IL-6, and TNF-α as compared to that from control cells, which were notably decreased by SHP-1 overexpression (Figure 4a). Additionally, western blot analysis showed that CES exposure led to an increase in the protein level of P65 phosphorylation compared to the control group, which was decreased by SHP-1 overexpression (Figure 4b). These data suggested that SHP-1 could attenuate CES-induced inflammation in 16HBE, possibly through modulating P65 phosphorylation.

### 3.5 SHP-1 inhibits activation of the PI3K/AKT pathway

**Figure 5:**
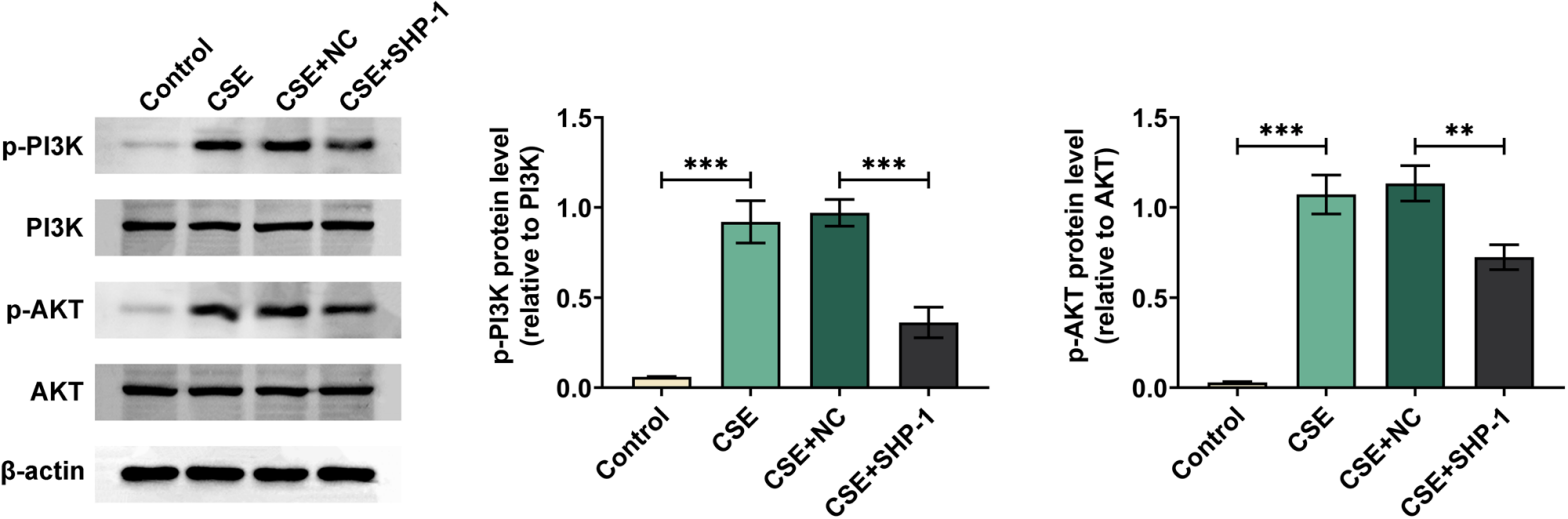
SHP-1 inhibited activation of the PI3K/AKT pathway. The protein expression of phosphorylated PI3K and AKT and total PI3K and AKT was examined by western blot. **p < 0.01, ***p < 0.001.

Finally, the effect of SHP-1 overexpression on the PI3K/AKT pathway in CES-treated 16HBE cells was studied. As shown in Figure 5, the CES group had increased phosphorylation levels of PI3K and AKT compared to the control group. However, the raised PI3K and AKT phosphorylation levels in 16HBE cells stimulated by CES were markedly reduced by SHP-1 overexpression. These data indicated that SHP-1 inhibited the CES-induced activation of the PI3K/AKT pathway in 16HBE cells.

## 4 Discussion

The dysfunction of the airway epithelium, a first barrier of defense in the respiratory system, induced by CSE is an early event in COPD [18,19]. In this study, we investigated whether SHP-1 is involved in suppressing CSE-induced 16HBE cell injury by treating 16HBE cells with 5% CSE for 24 h. The results showed that CSE exposure decreased cell viability, increased apoptosis and migration, and induced EMT and inflammation in 16HBE cells, which were greatly reversed by SHP-1 overexpression. Furthermore, the data found that SHP-1 could inhibit the activation of P65 and PI3K/AKT pathways caused by CSE.

Apoptosis of epithelial cells is a main mechanism of COPD pathology [20]. Our data showed that SHP-1 overexpression could promote 16HBE cell activity and reduce cell apoptosis under CSE exposure. Airway remodeling is the main pathophysiological basis of airflow limitation in COPD [21,22]. Studies have indicated that noxious gases, such as CSE, can elicit oxidative stress and persistent inflammation in the airways and lead to the decline of the airway epithelial tissue barrier function and the initiation of EMT [23–25]. The adhesion between transdifferentiated epithelial cells exhibited a decrease, accompanied by alterations in the composition of cytoskeletal proteins and an increase in migration ability, thus resulting in excessive deposition of extracellular matrix, periepithelial fibrosis, exacerbation of parenchymal structure damage in the small airways affected by COPD, and consequent airflow restriction [26,27]. Ultimately, this process contributed to the progressive advancement of airway remodeling [28]. Therefore, inhibiting EMT becomes a promising strategy for COPD treatment. Herein, we also detected the expression of migration and EMT-related proteins, and the results indicated that SHP-1 overexpression reversed CSE-induced cell migration and EMT. These data suggested that SHP-1 may retard COPD development by reducing epithelial cell apoptosis and attenuating airway remodeling.

Inflammation is the key factor leading to COPD development [29]. On the one hand, they promote airway injury and induce cell apoptosis, and on the other hand, they may cause airway remodeling [30,31]. As a result of further examination in this study, SHP-1 appeared to decrease the secretion of pro-inflammatory factors in 16HBE cells treated with CSE. Inflammation is known to be driven mainly by the NF-kB pathway [32]. In addition, NF-kB was found to control and regulate EMT programs in cells [33,34]. Our data further showed that SHP-1 could inhibit CSE-induced P65 phosphorylation. These data suggest that SHP-1 may reduce the inflammatory response in CSE-stimulated 16HBE cells by suppressing P65 phosphorylation.

PI3K/AKT signaling regulates cell survival, proliferation, differentiation, apoptosis, and other biological processes [35]. A lot of evidence supports the activation ofPI3K/AKT pathway was involved in COPD. Zhang et al. reported that activation of the PI3K/AKT pathway can stimulate the pathological changes of COPD by increasing the expression of inflammatory factors [36]. Xu et al. found that PI3K/AKT/NF-kB pathways may be involved in improving airway remodeling by inhibiting airway inflammation [37]. Wang et al. confirmed that activation of the PI3K/AKT pathway contributed to airway wall thickening in COPD [38]. These studies suggested that inhibition of the PI3K/AKT pathway may be an effective strategy for treating COPD. As expected, our data found that SHP-1 greatly reversed the activation of the PI3K/AKT pathway induced by CSE. However, further investigation is needed to determine whether SHP-1 protected 16HBE cells from CSE-induced damage by inhibiting the activation of the PI3K/AKT pathway.

In conclusion, we provided evidence that SHP-1 was lowly expressed in COPD patients and CSE-stimulated 16HBE cells. Furthermore, overexpression of SHP-1 could regulate CSE-induced cell proliferation, apoptosis, migration, EMT, and inflammation in 16HBE cells, and these effects may be associated with the inactivation of the PI3K/AKT pathway. Our findings suggest that SHP-1 may alleviate COPD by inhibiting airway inflammation and remodeling.
